# Risk Assessment of Surgical Interventions Performed on Non-Infected Patients During COVID-19 Pandemic

**DOI:** 10.7759/cureus.11682

**Published:** 2020-11-24

**Authors:** Anıl Demiroz, Servet Aydin, Can Ege Yalcin, Hakan Arslan

**Affiliations:** 1 Department of Plastic, Reconstructive and Aesthetic Surgery, Istanbul University-Cerrahpasa, Cerrahpasa Faculty of Medicine, Istanbul, TUR

**Keywords:** maxillofacial surgery, plastic surgery, preoperative testing, covid-19

## Abstract

Background

A number and a variety of surgical interventions were highly affected by the novel coronavirus disease (COVID-19) outbreak. Most of the elective operations were discontinued with the fear of exacerbating the disease in patients and spreading it among healthcare professionals.

Objective

The objective of this study was to report postoperative rates of COVID-19 in patients who underwent emergency and urgent surgery during the pandemic and to determine a safe algorithm in order to propose an ideal approach for surgeries.

Patients and methods

A total of 162 patients being operated upon emergency or urgent causes between March 11 and May 31 2020 were included in the study. Safety measures advised by the World Health Organization were applied. The patients' operative data and postoperative COVID-19 status were recorded and statistically evaluated.

Results

Surgical interventions were required for skin cancer, upper extremity trauma, soft tissue infections, maxillofacial trauma, lower extremity trauma and other causes. Local anesthesia was used for 127 patients (78.4%). General anesthesia was used for 28 patients (17,3%). Two of 162 patients contracted COVID-19 postoperatively on days 15 and 21, respectively. No statistical significance was found between surgery and anesthesia types regarding COVID-19 risk.

Conclusion

It appears that emergency and urgent surgeries can be performed safely. However, this relies upon adequate safety measures being taken with regards to screening for COVID-19 antigen positivity in patients preoperatively. Further evidence is required to determine the safety of elective surgeries.

## Introduction

Severe adult respiratory syndrome (SARS) was first described in 2002 as a lethal disease. It was caused by a subtype of coronavirus and resulted in a lower respiratory tract infection with respiratory failure. Within the spectrum of coronavirus related diseases of humans, the 2002 SARS infection almost reached a pandemic state, infecting 8,098 people causing 744 known deaths [1]. Two decades later, a related virus strain started an infection that evolved into a pandemic. Since the beginning of the infection in November 2019 until June 2020 it has already reached 10,000,000 cases worldwide.

The coronavirus family was originally described in the 1930s as a zoonotic pathogen that can cause respiratory tract infections in chickens, bats, mice and various other animals. Zoonotic sources of this virus are warm-blooded vertebrates. The first reported cases of human respiratory tract infections with coronaviruses were from the 1960s [2]. While infections in some animal species (such as chickens) have a bad prognosis with mortality rate of 40% to 90%, human infections were mostly mild. Most coronavirus subtypes like NL63, 229E, HKU1 and OC43 cause common cold-like infections in humans. However, the recently described severe adult respiratory syndrome-coronavirus 1 (SARS-CoV1), Middle Eastern Respiratory Syndrome-Coronavirus (MERS-CoV) and severe adult respiratory syndrome-coronavirus 2 (SARS-CoV2) can cause lethal lower respiratory tract infections in humans [3]. Treatment of these infections requires supportive therapies, hospitalizations and, in some instances, advanced ventilation strategies.

Pandemic

In November 2019, a series of cases who were diagnosed with SARS were reported from the Wuhan Province of China. A new strain of the well-known SARS-coronavirus (SARS-CoV) was thought to be the culprit behind this new disease [4]. The newly described SARS-CoV (named as SARS-CoV2) was different from its 2002 counterpart. The SARS infection spread throughout the whole globe at an accelerated pace causing a pandemic state in months. Nearly all the nations worldwide, the World Health Organization (WHO) and its dedicated committees have taken countermeasures to prevent the spread of the new pathogen [5]. The WHO named the new pandemic as coronavirus disease 19 (COVID-19). All the medical facilities and personnel available worked hard for the increasing numbers of COVID-19-related hospitalizations. Inpatient clinics were converted into pandemic wards and medical personnel were repositioned in most countries. Surgery clinics and surgical personnel were redistributed according to the quarantine protocols also. As a natural result and a preventive measure, all elective and non-urgent surgeries have been postponed without a guarantee of ever doing them anytime in the recent future.

COVID-19 in Turkey

In Turkey, the first COVID-19 case was reported on March 11, 2020. Patient number reached 150,000 in three months. As the burden increased and emergency departments became insufficient, most of the available inpatient clinics immediately became pandemic wards and medical personnel were also repositioned. Although surgery clinics were converted into pandemic wards, they still had to carry out their routine somehow. As in other parts of the World, all elective and non-urgent surgeries have been postponed. In our hospital, there was an initial stall period for the first few weeks of outbreak. In this period, scheduled surgeries were performed and outpatient clinics continued with the burden of approximately 80 patients a day routine. After restrictions were applied and safety measures were taken, all elective surgeries were stopped and outpatient services were limited to oncological admissions, acute trauma patients and chronic wound care follow-ups.

The aim of this study was to evaluate the incidence of COVID-19 and to determine whether it is safe to perform surgery with adequate measures in patients who had undergone inevitable surgeries such as skin malignancies, maxillofacial fractures, acute hand injuries and soft tissue infections.

## Materials and methods

This study was conducted after the approval of the Local Clinical Ethics Committee and the Ministry of Health in accordance with the Declaration of Helsinki. Records of all emergency and urgent surgeries performed in our department from March 11 2020 to the end of May were obtained. Emergency surgeries were performed for necrotizing soft-tissue infections, acute hand traumas and maxillofacial fractures. Oncological surgeries were performed on all patients with a skin malignancy. These surgeries included biopsies performed at outpatient clinics and surgeries performed in the operating room under local, regional or general anesthesia. A total number of 162 patients operated on were enrolled in this study. All the patients were followed till the end of June. Minimum follow-up period of the last enrolled patients was one month. There were no standard protocols for testing COVID-19 preoperatively until the beginning of the May rather than patients’ history and physical examination. During this period 81 patients were operated on and surgeries were performed with all personal protective equipment worn as advised by WHO. After this period, appropriate tests and preoperative workups advised by the government and hospital’s advisory committee were performed [6]. The advised workups were COVID-19 RNA polymerase chain reaction (PCR) for respiratory samples collected 48 hours before surgery for all patients and low dose radiation thorax computerized tomography (CT) scan for those with suspicion of COVID-19 pneumonia or known pulmonary disease. COVID-19 respiratory samples were collected preoperatively from all patients regardless of the anesthesia type excluding the outpatient clinic procedures. CT scans were performed only when indicated.

**Figure 1:**
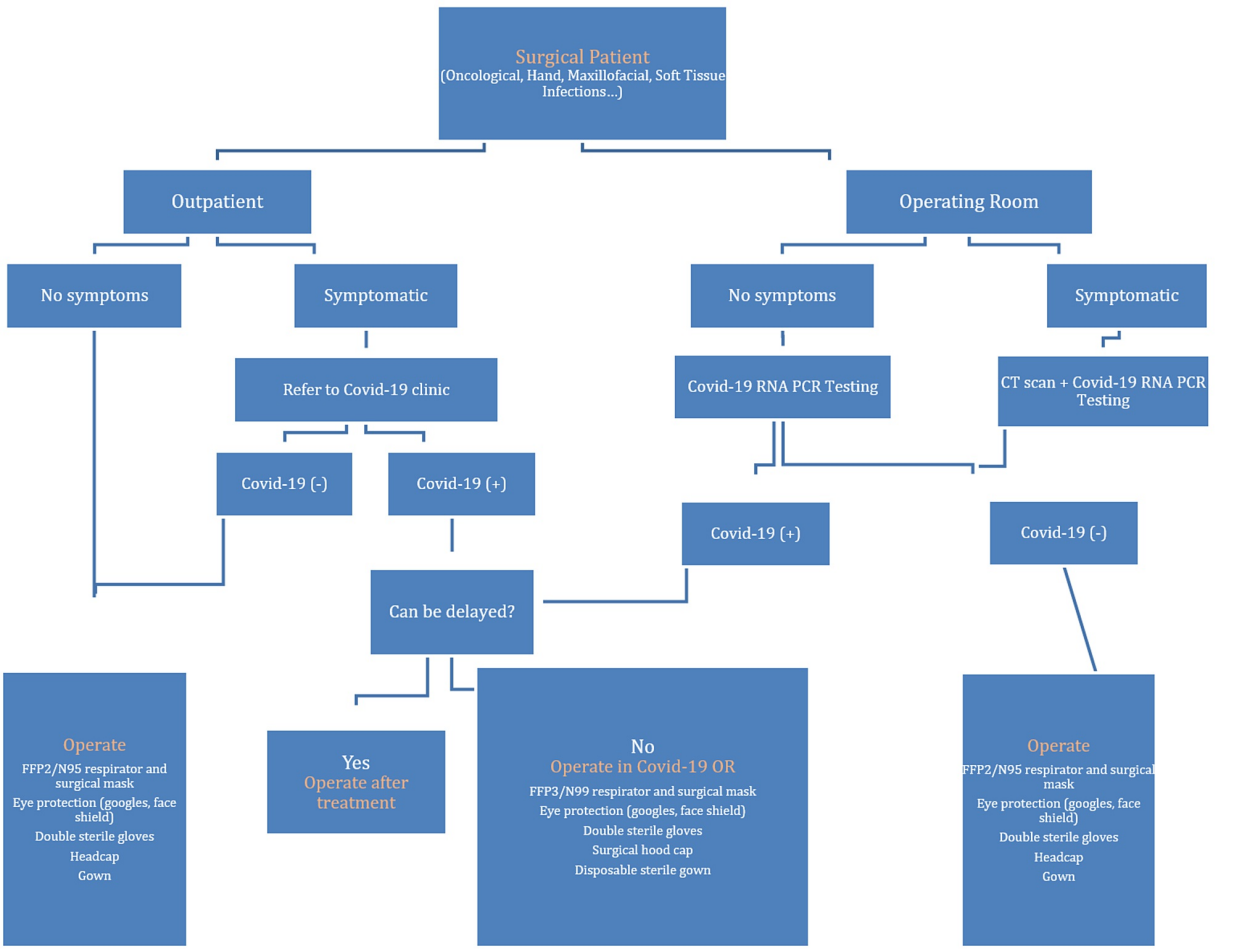
Algorithm for surgical preparation and preventive measures

Outpatient clinic procedures were performed after questioning for COVID-19 symptoms or close contact with COVID-19 positive persons. All the patients were educated about the risk of contracting coronavirus during the surgery, signed an informed consent form and underwent the surgery while the surgical team had safety glasses and double surgical gloves on. Patients with suspicious symptoms were referred to COVID-19 outpatient clinic for further assessment. While preparing the patients for surgery in the operating room, all the staff wore double examination gloves, protective gown, safety glasses, FFP2 or N95 respirators plus standard surgical masks. Surgeries were performed for patients that were negative for COVID-19 while applying safety measures determined by WHO and the Turkish Society of Plastic, Reconstructive and Aesthetic Surgeons (TSPRAS). Tracheal intubation processes were performed by Anesthesiology staff following the rules suggested by our hospital's advisory committee, which required all staff except for the anesthesiologists to leave the room. Although we did not operate on any patient with preoperatively diagnosed COVID-19, surgery of these patients was advised to be performed in an isolated operating room with enhanced personal protective equipment worn and the patients were transferred to pandemic wards after the surgery in our hospital. Figure 1 outlines the preventive measures taken for each type of setting.

Patients’ operative data including type of surgery and anesthesia, preoperative diagnosis and demographic properties were recorded. Patients were phone called on 15th and 30th days postoperatively to question for COVID-19 symptoms. Patients' recordings about COVID-19 were also checked on the Public Health Management System (PHMS). It is aimed to determine the effect of anesthesia and surgery type on COVID-19 incidence among the patients operated on, separately. Statistical significance was accepted with p-value < 0.05 and calculated using chi-square test among the groups determined. Statistical analysis was performed on Stata Version 13.1 (StataCorp., College Station, TX).

## Results

A total of 162 patients were operated between March 11 and May 31 at our institution with 99 of them being males and 63 of them being females. Age ranged between two years and 86 years with a mean of 45.9. Sixty-five patients were operated on at the outpatient clinic and the remaining 97 patients were operated on in the operating room. Distribution of anesthesia types for 162 patients is shown in Table 1.

**Table 1:** Distribution of anesthesia types

Anesthesia Type	Frequency (%)
Local Anesthesia	127 (78.39)
General Anesthesia	28 (17.28)
Infraclavicular Block	2 (1.23)
Combined Spinal-Epidural	2 (1.23)
Sedation	1 (0.62)
Spinal Block	2 (1.23)
Total	162

Operations performed for soft tissue infections (31.5%), melanoma and non-melanoma skin cancers (29%) and upper extremity trauma (22.8%). All outpatient procedures were performed under local anesthesia and included local debridement of soft tissue infections, which constituted the majority of the cases (53.8%), followed by excision of cancerous skin lesions (27.7%). 97 patients were operated on in the operating room, with the most common etiology being upper extremity trauma (38.1%). All patients were COVID-19 RNA PCR negative preoperatively. CT scan was performed for 22 patients who had symptoms for COVID-19 and with known pulmonary disease. All CT scans resulted in negative for COVID-19 pneumonia.

In the operating room, local anesthesia was used in 62 patients, the majority of which were upper extremity trauma procedures, while two patients required an infraclavicular block. In 28 patients, general anesthesia was used. Four patients were operated due to lower extremity trauma or lower extremity soft tissue infections under spinal or combined epidural blocks. One patient was operated using sedation and local anesthesia for excision and flap procedure for skin malignancy.

Maxillofacial trauma surgeries consisted the majority of the seven patients requiring oral interventions, of whom four underwent open reduction and internal fixation and two underwent intermaxillary fixation. The remaining one was orthognathic surgery. This patient was operated in the first week of the outbreak which was before the restrictions for elective surgery. Table 2 reveals the distribution of surgical indications.

**Table 2:** Distribution of patients according to surgical indications and admission type ORIF, open reduction and internal fixation; IMF, intermaxillary fixation

Categories of Surgery		Admission Type			Total
		Inpatient	On-call Surgery	Outpatient Clinic	
Soft Tissue Infection		15	2	35	52
Skin Cancer		27	0	21	48
Upper Extremity Trauma		16	22	3	41
Intraoral		7	0	0	7
	Maxillofacial Trauma ORIF	4	0	0	4
	Maxillofacial Trauma IMF	2	0	0	2
	Orthognathic	1	0	0	1
Other		6	0	8	14
Total		71	24	67	162

According to the follow-up questionnaire, two patients had shown mild symptoms of COVID-19 at postoperative days 15 and 21, respectively. Both the patients had undergone biopsy for suspected skin cancer in the outpatient setting during March. After the onset of symptoms, COVID-19 diagnosis was confirmed on PHMS. Both patients did not undergo preoperative testing for COVID-19. First patient was a 45-year-old female presented with fever and cough on 15th day postoperatively to the emergency department. Testing for COVID-19 RNA PCR was positive. No CT scan was performed for this patient. Patient was a nurse repositioned at a pandemic ward. She was prescribed suggested COVID-19 treatment for one week and was isolated at home for 14 days [7]. Symptoms resolved after treatment. The other patient was a 35-year-old male who is also a hospital staff. Patient was admitted to the emergency department with fatigue and fever symptoms 21 days after the operation. Initial COVID-19 RNA PCR testing was negative and the CT scan was also negative for COVID-19 pneumonia. Due to the proceeding symptoms of COVID-19 and previous history of close contact with a COVID-19 positive person, the patient was diagnosed as probable COVID-19 and was given a one-week treatment. Patient was isolated at home for 14 days and symptoms resolved completely. PCR testing at the end of the treatment was negative.

Symptom questioning and PCR testing were done at the end of study for our surgical, anesthesiology and operating room staff, none of whom reported any symptoms or tested positive with PCR.

Our results showed that two positive patients required treatment for COVID-19 from all 162 patients (1.23%). Positive result rate among the local anesthesia patients was 1.57%. No patients tested positive in the general anesthesia, regional anesthesia, spinal/epidural block and sedation groups. There was no statistical significance between the anesthesia type groups (p=0.455). Positive cases had undergone skin cancer surgery. No positive results were present among the other surgery types. There was no statistically significance between the surgery types (p=0.082).

## Discussion

As the COVID-19 crisis happened and impaired the health systems worldwide nearly all the elective surgeries ceased in a stepback fashion. To bear the burden, operating rooms were converted into intensive care unit rooms for critically ill patients. Most of the hospitals kept a few operating rooms for emergent procedures only. Patients waiting for operations were accumulated. As the numbers increased the surgeries were continued after a brief period of cessation. Emergency admissions have shown regression in the very first weeks also. Emergency admissions were increased slowly after on.

Upper extremity trauma was one of the leading causes of surgeries in our study. Upper extremity trauma admissions to our hospital have shown a decline during the COVID-19 pandemic and we continued to perform emergency surgeries. Most injuries were due to accidents at home. Frequency of the trauma at work showed a significant decline in our study also reported by Garude et al. Hands, solely are a vessel of contamination, and disinfection of hands regularly have shown to be effective in limiting the spread of COVID-19 [8]. Although there are no reported cases of a healthcare worker got infected by upper extremity examination, extra care must be taken when assessing the upper extremity injury, especially the hand. Although Naturally, trauma patients were not expected to clean their hands before the accident, they also cannot wash their hands easily after the injury so it is important to decontaminate hands after the admission to the hospital and standard safety protocols should be applied [9]. In our practice, we use isotonic serum irrigation first for cleaning the wound from debris and blood. Affected extremity was then scrubbed with %10 Povidone Iodide solution and placed on the sterile hand table for surgery.

Skin malignancy surgery was important in our study. Skin malignancy entities require urgent surgery because of their aggressive nature. We performed surgery for all types of basal cell carcinoma, squamous cell carcinoma and malignant melanoma. Considering the restrictions and limited outpatient clinic admissions, delayed presentations were commonly observed. Fear of the COVID-19 mortality among older people can further delay the admissions because most of the skin malignancy patients were in the risk group of age for COVID-19, especially in the head and neck region. Thus when performing surgery, it is best to select the appropriate reconstruction method both for reducing the operative time and hospitalization stay time postoperatively. Reconstruction after resection of skin malignancies is especially important in the head and neck region. Pushing the maximum conservative treatment option within the reconstructive ladder can shorten the time of surgery, hospitalization and further visits to hospital and thus reduces the risk of transmission. It is appropriate to use the pedicled and safer flaps instead of free flaps for reconstruction [10]. Tracheal intubation time is important for both the patient and the operating staff because longer mechanical ventilation time produces more aerosols which can lead to increased risk for COVID-19. When possible, local or regional anesthesia techniques should be selected to reduce the risk of transmission via aerosol generated procedure. Most of the upper extremity trauma and skin malignancy surgery can be performed without general anesthesia. However, it is important to detect the cases which can be quicker and comfortable with general anesthesia.

Oral and maxillofacial surgery is another risk factor for transmission of COVID-19. One of the first doctors to lose his life was an otorhinolaryngologist [11]. Oral secretions and droplets are very contagious and the disease transmits via direct exposure as well as contact. Therefore aerosol-generating examination methods should be performed cautiously and best avoided when possible. Physical examination of oral cavity and facial region should be performed with agility and full personal protection equipment [12]. Holmes et al. recommended using conventional intermaxillary fixation (IMF) for maxillary and mandibular fractures during COVID-19 pandemic to reduce the risk of transmission [13]. When possible this option should be preferred however complex and most comminuted fractures require open reduction and internal fixation (ORIF). We performed four ORIF and two IMF procedures. One of the two IMF patients was an eight-year-old female with bilateral condylar mandible fracture. Patient went to the operating room and required anesthesia for both implanting and extracting the screws for IMF. The other surgery was orthognathic surgery for type 3 malocclusion. This surgery was performed in the early days of outbreak when there was no cancellation of surgeries. So this surgery shouldn't be accepted as mandatory but still kept in our study because it was a high-risk surgery that was performed after the outbreak. Oral or facial procedures should be accepted as high-risk surgeries for COVID-19 transmission due to the virus's way of transmission. Nevertheless, no patient or staff showed positive results in this group of surgery.

Whether necrotizing or not, soft tissue infections including diabetic foot infections are another important aspect of the pandemic because of the possible late presentation of the patients due to limited outpatient clinic admissions. When possible, preventive measures and small interventions were performed in the outpatient setting in order to avoid advancement of the infection. Due to the higher prevalence of comorbidities among this patient population, they had worse prognosis of COVID-19 infection. Therefore, a balance should be kept where the patient is still closely followed up for infection while necessary interventions are performed and the time spent at the hospital is minimized.

Telemedicine methods can be used as a tool for limiting the hospital visits especially among old, comorbid patients who are at high risk for COVID-19 [14]. Regarding the follow-ups of patients, telemedicine can be a very effective method for specialties like Plastic Surgery and Dermatology in which the inspection is very important. Regular photographing of chronic wounds and information about wound beds can guide the surgeon to come up with a unique plan for wound care. However, care should be taken on the time to assess the patient in person for a timely intervention.

When questioned for COVID-19, we learned two of our patients had contracted the disease. Both of these patients had undergone a biopsy for skin cancer in an outpatient setting. Both of them were lacking preoperative laboratory testing for COVID-19 and were admitted during March when there was no standard protocol. Patients may have contracted the disease from outpatient clinics where it is obvious that many more patients and people visit than the operating room. Since both patients are hospital staff and the female patient actively worked at a pandemic ward after surgery, it is also possible that the patients contracted the disease not because of surgery-related visit to hospital.

Studies about the safety of performing specific types of surgery during COVID-19 pandemic are limited. One of the first reports of continued elective abdominal surgery outcomes by Lei et al. have shown worrying results [15]. Authors reported a series of 34 patients, all of whom developed COVID-19 pneumonia, 44.1% of which required transfer to the intensive care unit. Their mortality rate was 20.5%. All of the patients were known to travel the city of Wuhan, where was the starting point of COVID-19. However the patients didn’t show any symptoms before, there was also no preoperative testing for them. Authors concluded their patients were in the incubation period of COVID-19 and surgery in this period was most likely to be fatal. Aminian et al. also reported that elective surgeries on patients with undetected COVID-19 may result in a high fatality rate [16]. These results demonstrated the importance of preoperative testing before any type of surgery to be performed.

COVID-19 RNA PCR testing routinely and CT scan of thorax when indicated were suggested as a preoperative laboratory testing and performed in this study. Several studies concluded that COVID-19 RNA PCR respiratory sampling can be false negative up to %40 depending on the site where the samples were collected [17]. Broncho aspiration technique shown to be the ideal way to reduce false-negative ration however it is impractical to use preoperatively [18]. Combined oral and nasopharyngeal swab technique was used in our study routinely to collect for samples as indicated by WHO. CT scan of thorax can be used to overcome the handicap of PCR testing but it comes with the disadvantage of radiation. Also limited number of retrospective studies regarding the diagnostic use of CT for COVID-19 remains unproven [19]. In our study, all performed CT scans were negative for COVID-19 pneumonia. Apart from previously mentioned two patients, none of the patients in this study have shown any symptoms of COVID-19. Our results demonstrate that procedures either performed at an outpatient setting or in the operating room can be safely performed as long as the standard safety measures are followed. We did not observe any statistically significant results either among anesthesia or surgery types. Therefore, not only emergent surgeries, but also surgeries for cancer, non-emergent trauma including maxillofacial traumas and soft tissue infections can be safely undertaken regardless of the anesthesia type. However, to continue for elective surgeries, still more evidence is required.

Major limitation of our study was its timing. Since there was no standard preoperative protocol until the beginning of May, patients could not be proven laboratory negative for COVID-19. Only two patients tested positive for COVID-19 during this period. All patients except the aforementioned ones, COVID-19 RNA PCR testing showed negative results and no symptoms developed during the follow-up period. According to these results, preoperative COVID-19 RNA PCR testing could be an efficient preoperative test when performed 48 hours before surgery. Another limitation of our study is that we had no patients with preoperatively diagnosed COVID-19, either by PCR or computerized tomography. We therefore could not observe the effect of surgery on the clinical outcome of these patients or its effect on the infectivity among other patients or healthcare workers.

## Conclusions

COVID-19 pandemic did not only affect the patients who contracted the disease, but also patients who require urgent or emergent surgical treatment. In this article, we aimed to present our series of patients who were surgically treated during the pandemic and followed up for postoperative COVID-19 prevalence, which demonstrated PCR-testing as an effective method for screening and with adequate preventive measures these procedures can be undertaken. However, we want to emphasize that further studies are required to confirm the possibility to move on with the elective surgeries.
